# Sonographic Features of a Tuberculous Cold Abscess: A Case Report and Literature Review

**DOI:** 10.24908/pocus.v8i1.15831

**Published:** 2023-04-26

**Authors:** Amir Alzarrad, Salman Naeem, Serena Rovida

**Affiliations:** 1 Emergency Department, Barts Health NHS Trust London United Kingdom

**Keywords:** ultrasound, extrapulmonarytuberculosis, osteomyelitis, coldabscess, Point of Care Ultrasound(POCUS)

## Abstract

The use of point of care ultrasound (POCUS) to aid diagnosis of tuberculosis has been investigated in countries where concomitant endemic prevalence of HIV increases the incidence of extrapulmonary tuberculosis (EPTB). In such cases, using a focused assessment with sonography for HIV-associated tuberculosis (FASH) scan has found to be immensely advantageous as a rapid diagnostic tool in low resource settings where other imaging modalities are scarce. The prevalence of EPTB in immunocompetent patients in industrialised countries is growing. Since EPTB can manifest itself in almost any part of the human body, symptomatic patients present with constitutional and non-specific symptoms. In our case, a 44-year-old male presented to the emergency department (ED) with a 3-month history of left-sided chest pain and swelling of the chest wall. Clinical examination revealed a swollen and tender lump above the left first rib. Palpation of the thoracic (T7) vertebral body demonstrated localised pain. POCUS showed a collection of heterogenous material with fluid content and specks of hyperechoic ‘ring-like’ structures. Further investigations led to the diagnosis of EPTB. The patient was admitted and treated for EPTB where he went on to make a full recovery. This case report highlights the role of integrating POCUS in clinical examination of patients with suspected EPTB, which can expedite its diagnosis and management.

## Introduction

Tuberculosis (TB) remains a global public health concern; most notably in endemic countries where there is a rise in its incidence. Although primary pulmonary involvement accounts for the majority of TB cases, extrapulmonary tuberculosis (EPTB) is rapidly growing in high income countries. Anatomical sites for EPTB include pleura, joints, bones, lymph nodes, urogenital tract and meninges. Constitutional symptoms such as fever, weight loss, night sweats, anorexia are common but typically unspecific in presenting patients. 

The use of point of care ultrasound (POCUS) to aid diagnosis of tuberculosis has been investigated in countries where concomitant endemic prevalence of HIV increases the incidence of EPTB. Co-existence of TB and human immunodeficiency virus (HIV) epidemics has created a rise of EPTB cases. In such cases, using a focused assessment with sonography for HIV-associated tuberculosis (FASH) scan has been found to be immensely advantageous as a rapid diagnostic tool in low resource settings where other imaging modalities are scarce 1. This case report describes the use of point POCUS in the diagnosis of cold abscess and osteomyelitis.

## Case description

A previously fit and well Pakistani-born 44-year-old male patient presented to the Royal London ED with a 3-month history of constant left-sided chest pain and swelling of the chest wall. He reported unintentional weight loss (approximating 15 kg over 2 months) associated with an enlarging left-sided chest wall mass, night sweats and postural dizziness. He denied cough, haemoptysis and dyspnoea. His initial vitals recorded a respiratory rate of 17 breaths/min, heart rate of 137 beats-per-minute, blood pressure of 154/106mmHg and a temperature of 37.2 °C. Clinical examination revealed pain on palpation of the thoracic vertebral body at the level of T7. There was no associated or regional lymphadenopathy. Blood investigations revealed microcytic anaemia (HB of 117 g/L and MCV 75.7 fL), thrombocytosis (platelets 410 10^9^/L) and lymphocytopenia (0.7 10^9^/L). The patient’s HIV status was negative. 

**Figure 1:**
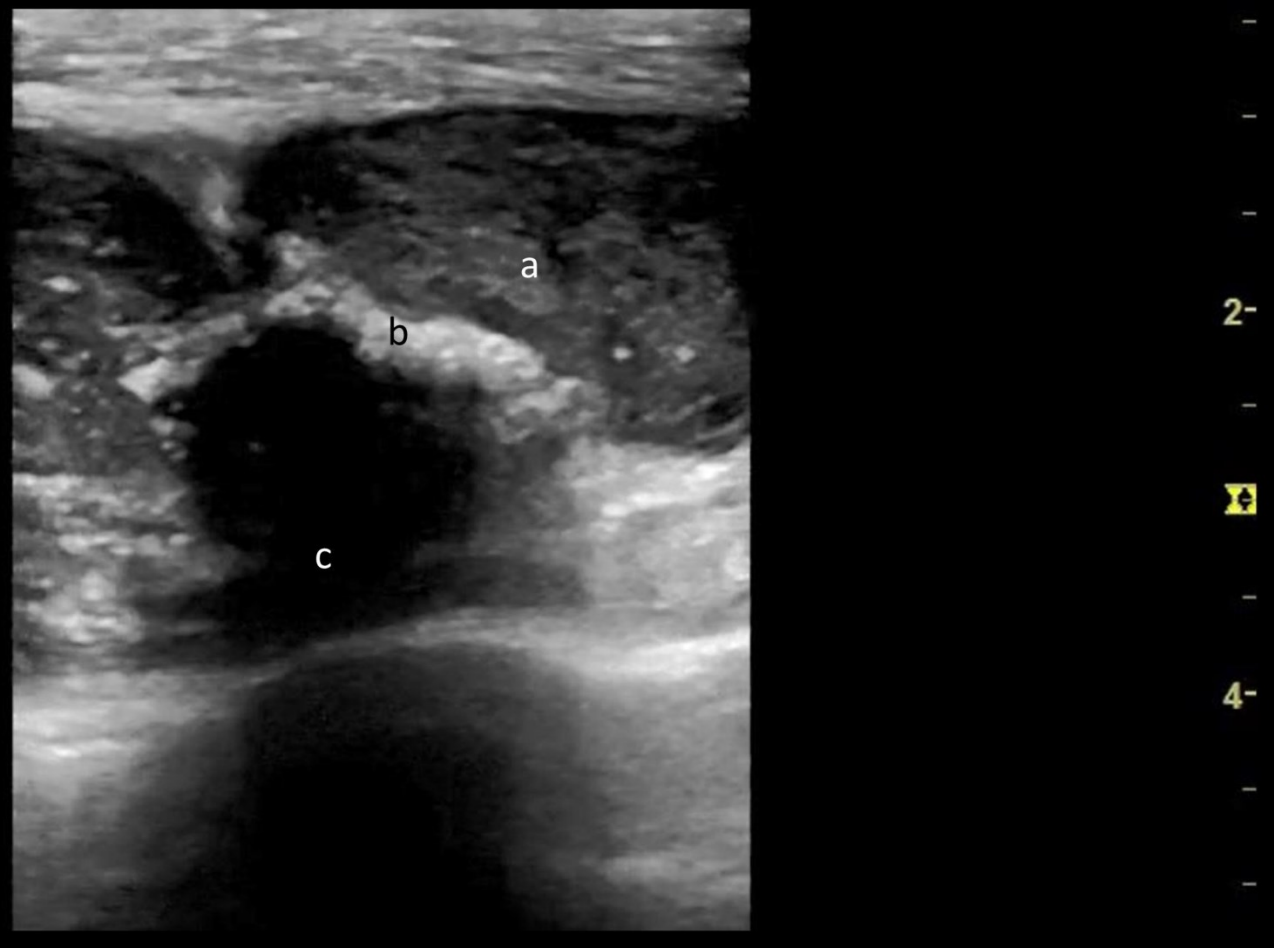
A sonographic image of the cold abscess using a 12 MHz linear probe showing heterogenous fluid collection (a) in the pectoralis major on the left hemithorax. Thickened periosteum of the first rib (b) with break in the cortex representing osteomyelitis. There is posterior acoustic shadowing below the first rib (c).

**Figure 2:**
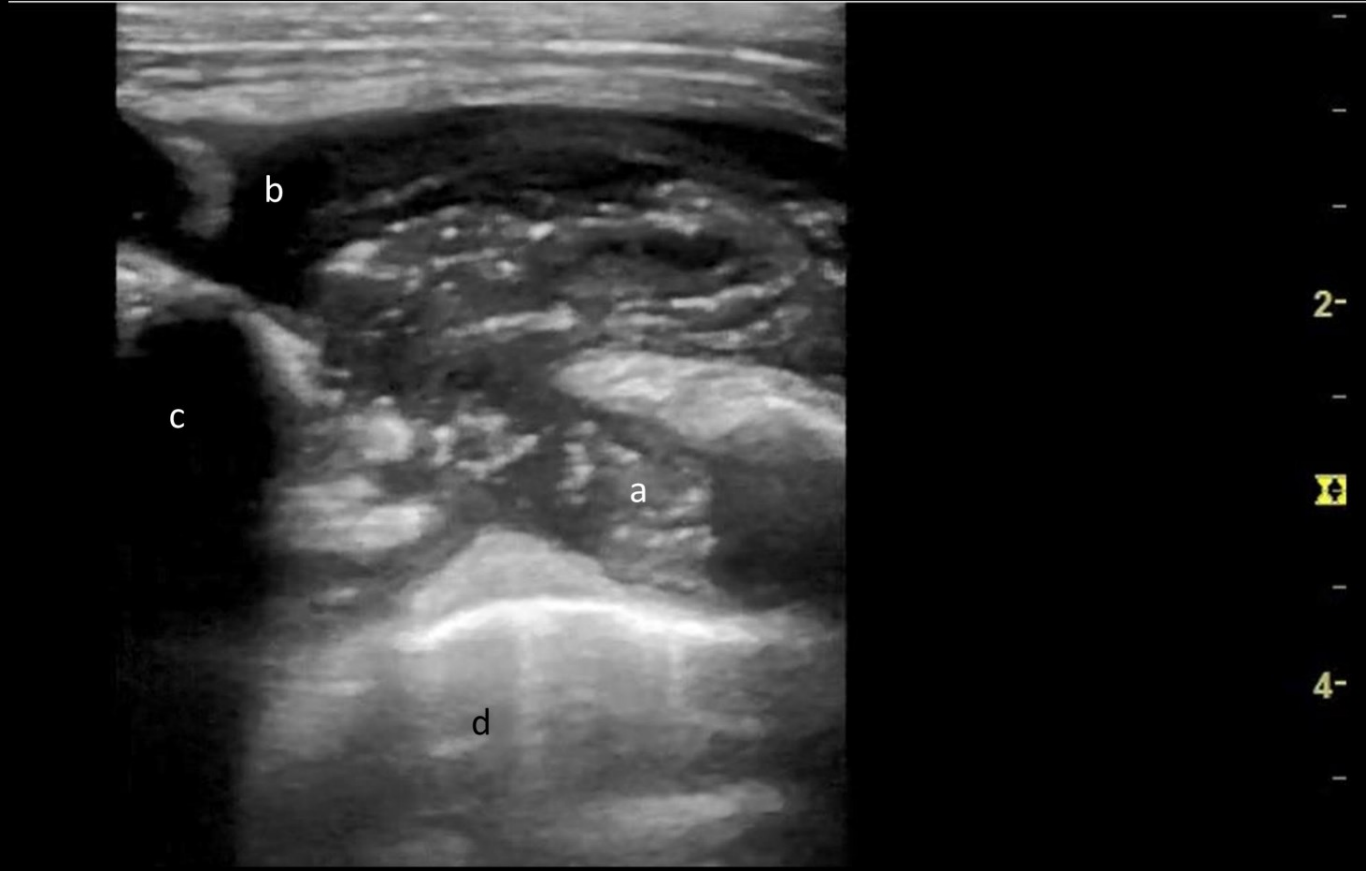
A sonographic image of the cold abscess using a 12 MHz linear probe showing fluid collection of mixed echogenicity. There are ‘ring lesions’ (a) with hyperechoic irregular margins and relatively hypoechoic centre which might represent caseous lymph nodes. There is anechoic fluid (b) and posterior acoustic shadow of the first rib (c) with under lying lung (d).

**Figure 3:**
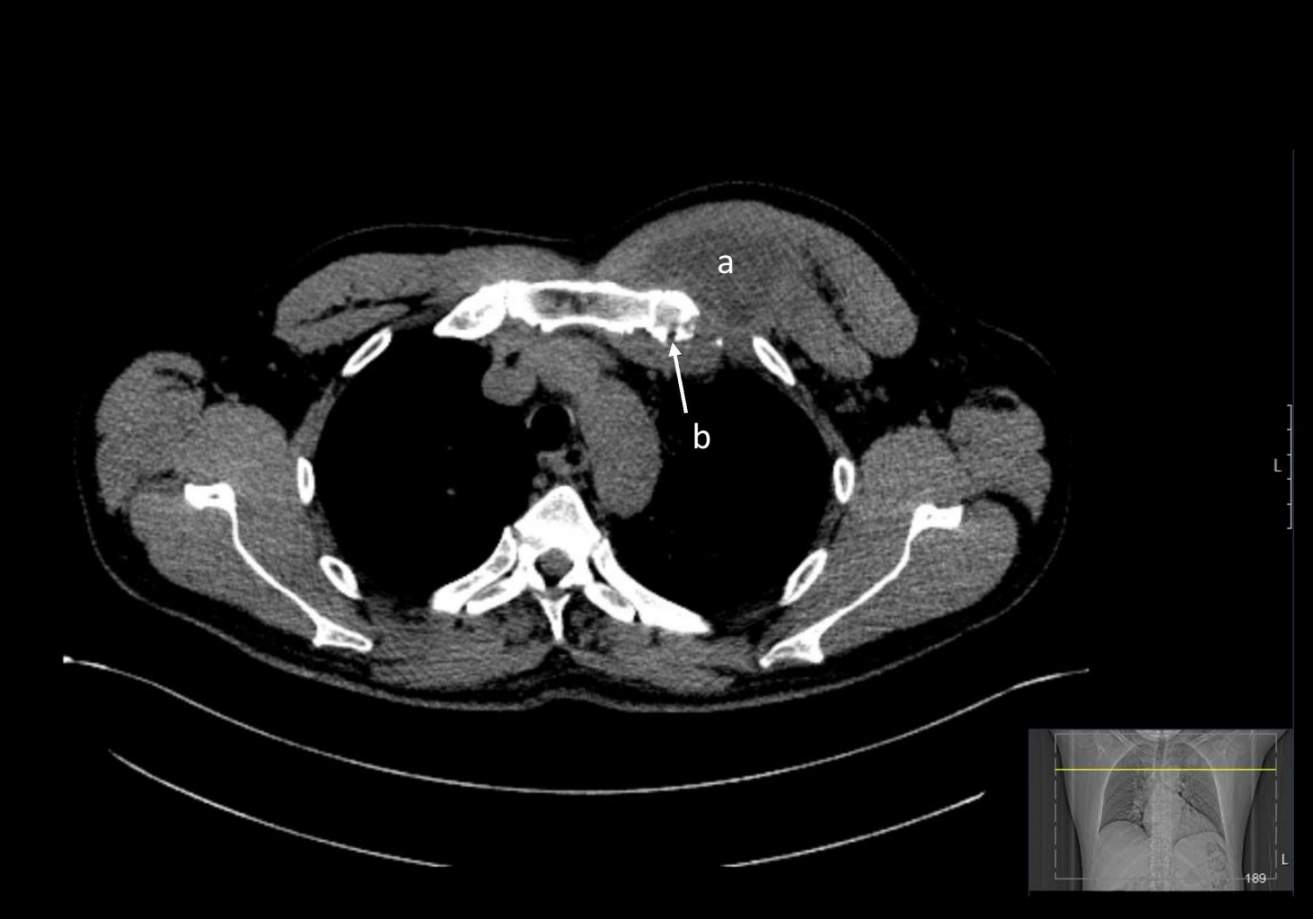
Axial view of the plain CT thorax showing low density collection in the pectoralis major muscle of the left hemithorax (a) with underlying costochondral junction showing break in cortex (b).

POCUS performed by an ED physician showed a collection of heterogenous material with fluid content (Figure 1) along with hyperechoic, thickened periosteum of the left first rib at the level of the pectoralis major muscle (Figure 2). Specks of hyperechogenic material with ‘ring-like’ structures were also noted. These structures had a hyperechoic, ring-shaped border surrounding a hypoechoic centre. Chest X-ray showed an abnormal opacification within the left apex and upper zone. Subsequent computerised tomographic (CT) scan of his thorax CT confirmed a 66 mm x 30 mm left upper chest wall abscess involving left costochondral junction and engulfing the distal left subclavian vein (Figure 3).

The patient was admitted under the medical team and treated for osteomyelitis with broad spectrum antibiotics and a high suspicion of EPTB. Aspirate from the abscess grew Mycobacterium tuberculosis, confirming the diagnosis. Full body CT scan further revealed thoracic and lumbar disseminated spinal TB alongside destruction of the left first rib at the T1 junction. He was started on anti-tuberculous medications and followed up by the TB outpatient and spinal clinics for a 12-month period. 

## Discussion

EPTB is gaining renewed interest especially in industrialised countries where the incidence rates have increased over recent years. In 2002 the proportion of tuberculosis infections manifesting as EPTB was estimated at 16.4%. By 2011, this has increased to 22.4%. In England, EPTB rates are currently amongst the highest levels recorded, accounting for ~40% of all TB cases 2. While the most common site of EPTB are lymph nodes, TB can infiltrate almost any part of the human body. Musculoskeletal involvement of EPTB accounts for 20–30% of EPTB cases, and vertebral bones remain the predominant site of infection 3. Tuberculosis of chest wall is an uncommon form of osteoarticular tuberculosis and accounts for 1-5% of bone tuberculosis 4. Due to the varied and nonspecific signs and symptoms of EPTB, it is difficult to make the diagnosis solely on history and physical examination. It thus requires specialist investigations, and a high index of suspicion in the appropriately identified patients. This potentially delays the diagnosis and its management. 

Tubercular abscesses is an uncommon presentation of EPTB and is mainly encountered in immunocompromised patients. Abscesses contain pus infected with microorganisms. Bacterial abscesses are typically warm, red, and tender to touch (characteristic signs of inflammation). However, a cold abscess lacks such inflammatory signs and is usually a sequalae of tubercular infection 5. Early diagnosis and treatment of cold abscess is essential to prevent peripheral spread.

In immunocompromised patients, the clinical presentation of EPTB is generic – typically consisting of weight loss, night sweats and fever. This makes the diagnosis of TB greatly challenging. Whilst the disease progresses, the risk of having EPTB manifestations also increases. Studies have shown a 45% - 56% prevalence of EPTB in patients with AIDS 6. Thus, incorporating POCUS as an adjunct to assist the diagnosis of TB using the FASH protocol is increasingly beneficial as it allows early identification and treatment of EPTB. FASH findings includes identifying pericardial, pleural and ascitic effusion, abdominal lymphadenopathy, and hepatic and splenic micro-abscesses 7. Sonographically, abscesses may appear as anechoic (coloured black on ultrasound) or hypoechoic (darker than their surrounding tissues) collections. Their shape is most often circular but with irregular, thickened walls. As an abscess further develops, the material within can form more hyperechoic foci and sedimentation and in some cases, gas may be present within the fluid collection 8. As the mycobacterium infection continues to progress, the nodules caseate, become denser and necrose which will provide a more heterogeneous echotexture 9. 

Ultrasonography can similarly image soft tissues and joints adjacent to the infected bone. This is because they can be used to visualise soft tissue abscesses, subperiosteal collections and joint effusions 10. In acute pathologies, periosteal reactions cause an elevation of bone periosteum. This causes the periosteum to become thick and oedematous, losing its sharpness 11. 

In this case, the sonographic and clinical findings of cold abscess along with constitutional symptoms were consistent with TB. POCUS of the cold abscess showed a fluid collection of mixed echogenicity along with ring-like structures with hyperechoic irregular borders and hypoechoic centres, representing caseating lymph nodes. These echogenic foci possibly represent caseating necrosis of lymph node and TB is one of the differentials. This case report highlights how integrating POCUS within clinical practice can allow a physician to provide a more detailed evaluation of patients and test a suspected hypothesis, which as a result allowed the patient to have an expedited diagnosis and management of his disease.

## Consent

Full consent by the patient is approved and attained.

## Conflicts of Interest

None.
